# Understanding the metabolome and metagenome as extended phenotypes: The next frontier in macroalgae domestication and improvement

**DOI:** 10.1111/jwas.12782

**Published:** 2021-03-24

**Authors:** Kelly J. DeWeese, Melisa G. Osborne

**Affiliations:** Molecular and Computational Biology Section, Department of Biological Sciences, University of Southern California, California, Los Angeles

**Keywords:** aquaculture, domestication, gene regulatory networks, macroalgae, marine, metabolome, metagenome, metagenomics, metatranscriptomics, phenotype, structural equation modeling

## Abstract

“Omics” techniques (including genomics, transcriptomics, metabolomics, proteomics, and metagenomics) have been employed with huge success in the improvement of agricultural crops. As marine aquaculture of macroalgae expands globally, biologists are working to domesticate species of macroalgae by applying these techniques tested in agriculture to wild macroalgae species. Metabolomics has revealed metabolites and pathways that influence agriculturally relevant traits in crops, allowing for informed crop crossing schemes and genomic improvement strategies that would be pivotal to inform selection on macroalgae for domestication. Advances in metagenomics have improved understanding of host–symbiont interactions and the potential for microbial organisms to improve crop outcomes. There is much room in the field of macroalgal biology for further research toward improvement of macroalgae cultivars in aquaculture using metabolomic and metagenomic analyses. To this end, this review discusses the application and necessary expansion of the omics tool kit for macroalgae domestication as we move to enhance seaweed farming worldwide.

## 1 | INTRODUCTION

The farming of marine macroalgae (seaweed) contributes to the international food, cosmetic, science, and pharmaceutical industries (Buschmann et al., 2017; FAO, 2020). Aquaculture of macroalgae has historically been almost exclusively an Asian economic enterprise—over 99% of annual production is based in Asia (Ferdouse, Løvstad Holdt, Smith, Murúa, & Yang, 2018). With increased globalization and the search for ecofriendly alternatives in the food and biofuel industries, aquaculture has grown outside of Asia as well. Recently, Europe and the Americas have seen an emergence of aquaculture farms, companies, and research facilities specializing in algae (Kim, Yarish, Hwang, Park, & Kim, 2017). Over the last two decades, world production of marine macroalgae has more than tripled, up from 10.6 million tons in 2000 to 32.4 million tons in 2018 (FAO, 2020). Although there are hundreds of known marine macroalgae species, 81% of algae production is composed of only a handful of brown and red macroalgae species (FAO, 2018). Aquaculture, particularly that of marine macroalgae, has much potential to expand globally onto different coastlines and harnessing new species. Furthermore, there are large economic and environmental gaps to fill, specifically in the realms of biofuel and food production (Duarte, Wu, Xiao, Bruhn, & Krause-Jensen, 2017; Guzinski, Ballenghien, Daguin-Thiébaut, Lévêque, & Viard, 2018; Pereira & Yarish, 2008; Sudhakar et al., 2018). Macroalgae are an especially attractive species for food and biofuel crops as they do not compete with agriculture for land or freshwater resources (Kim, Stekoll, & Yarish, 2019). From the growing pressure that climate change presents to agriculture, aquaculture has quickly gained global attention and many species of macroalgae are positioned to become significant marine crops.

### 1.1 | Brief review of marine macroalgae aquaculture

Seaweed farming, and its impact on aquaculture, has evolved over hundreds of years (Pereira & Yarish, 2008). It began as a practice of coastal harvesting, and the increasing use and value of seaweed in human food products has led to more direct farming of seaweed as a crop. In 2018, farmed seaweeds represented 97.1% by volume of the total of 32.4 million tons of wild-collected and cultivated aquatic algae combined (FAO, 2020). Although global production of farmed seaweed has experienced relatively slow growth in recent years, a renewed interest in sustainable foods, feeds, phycocolloids, and biofuel production has ignited groups in academia and industry to focus efforts on developing several species of macroalgae as commercial crops (Kim et al., 2019). In fact, over the last 30 years, several US federal agencies, including the Department of Energy (DOE), Department of Agriculture (USDA), and Department of Commerce’s National Oceanic and Atmospheric Administration (NOAA), have invested nearly 1 billion dollars into developing the United States as a leader in aquaculture (Kim et al., 2019; Love, Gorski, & Fry, 2017).

Seaweeds are extractive crops, meaning that they benefit the environment by removing waste materials such as nitrogen, phosphorus, and carbon (FAO, 2018; Kim, Kraemer, & Yarish, 2015). High levels of these materials can have negative consequences on coastal ecosystems by triggering, for example, harmful microalgae blooms. However, these materials can be absorbed by several species of seaweed and are in fact important nutrients to support proper growth and development (Buck, Nevejan, Wille, Chambers, & Chopin, 2017; Rose et al., 2015). In addition to being an environmentally friendly crop, seaweeds are increasingly being recognized for their abundance of nutrients including, but not limited to, iodine, iron, and vitamin A (FAO, 2020; Tanna & Mishra, 2019). Seaweeds contain micronutrient minerals (e.g., iron, calcium, iodine, potassium and selenium) and vitamins (particularly A, C, and B_12_) and are the only non-fish sources of natural omega-3 long-chain fatty acids (FAO, 2020). Beyond their applications in the human food industry, seaweeds have a diverse range of commercial applications such as additives in feeds, fertilizers, and cosmetics; production of alginate, agar, and carrageenan; pharmaceuticals; and biofuels (Buschmann et al., 2017; Kim, 2015; Kim et al., 2017; Pereira & Yarish, 2008).

### 1.2 | Modern farming in the United States and targeted breeding

In the 50 years between 1950 and 2000, the human population more than doubled, increasing in an unprecedented manner from 2.5 billion to 6 billion (Bongaarts, 2009). At the head of the population boom, the 1960s were fraught with concern about the ratio of food to population (Khush, 2001). This sentiment contrasts starkly with our reality today, where we struggle to minimize food waste in developed countries (Walia & Sanders, 2019). Agriculture was able to keep pace with the human population with its own productivity boom now called the “green revolution,” a period in which advanced breeding crop techniques were developed and crops were first genetically modified to increase yields (Khush, 2001). Since the green revolution changed the face of agriculture, entire fields of science have developed to further investigate the genes, variants, expression profiles, and metabolites involved in creating high-yield, superior crops.

The past two decades have seen genomics and other omics disciplines begin to dominate the study of agriculture. Many significant agricultural crops have had their genomes sequenced, allowing scientists to gain an understanding of the gene networks and pathways that determine crop success for targeted breeding approaches (Van Emon, 2016). Modern genomics techniques for improving agricultural crops include quantitative trait loci (QTL) mapping and genome-wide association studies (GWASs) (Huang & Han, 2014). QTL mapping identifies genetic loci that cause or contribute to specific phenotypes in crops, while GWAS identifies single-nucleotide polymorphisms (SNPs) associated with traits of interest (Huang et al., 2015; Murray et al., 2008). QTL mapping has been applied to crop improvement in agricultural breeding programs through marker-assisted selection (MAS), which utilizes polymorphic regions linked to specific alleles to detect and select for desirable alleles and traits in crop cultivars (Collard & Mackill, 2008). However, MAS is best able to detect high-effect genes or monogenic traits (Heffner, Sorrells, & Jannink, 2009; Xu & Crouch, 2008). The limitations of MAS can be overcome in part using advanced genetic methods that incorporate GWAS models into breeding, in a process known as genomic selection (Goecke, Klemetsdal, & Ergon, 2020). In genomic selection, SNPs are used as markers across the entire genome to estimate breeding values, a technique that has a far higher resolution than MAS, can help to detangle tightly linked traits, and is starting to model polygenic traits (Heffner et al., 2009). Microarray and RNA sequencing data, used to build transcriptomes, and expression profiles, as well as to conduct differential expression analyses, have also been instrumental in identifying significant genes and variants in agricultural crops (Ashikari et al., 2005; Van Emon, 2016). To review the use of molecular markers and genomic breeding programs in seaweed aquaculture, we refer the reader to Yong, Chin, and Rodrigues (2016) and Goecke et al. (2020). For a more recent review of genomic approaches for improving agriculture more broadly, we also point the reader to the review by Bohra, Chand Jha, Godwin, and Kumar Varshney (2020).

Even more recently, metabolomics and metagenomics data have been incorporated into analyses for crop improvement (Dixon et al., 2006; Gastauer et al., 2019; Lin, Lemay, & Parfrey, 2018; O’Donnell, Ross, & Stanton, 2020; Van Emon, 2016; Walker et al., 2016). Metabolomics provides insight into pathways involved in growth and other desirable phenotypes, while metagenomics reveals associated microbial species and putative functions that they could be performing for their crop hosts such as nitrogen fixation.

Although humans have utilized seaweed as a food source for hundreds of years, its presence in Western diets is concentrated in scattered coastal areas including Maine, Hawaii, and Alaska (Hunter, 1975; Kim et al., 2019; Mouritsen et al., 2013). The vast majority of global seaweed production is grown in Asia (FAO, 2018), and aquaculture is one of the fastest growing maritime industries in the United States, particularly around New England (Kim et al., 2019). With the growing accessibility of and technology for multi-omics applications (O’Donnell et al., 2020), this presents a timely opportunity for domestication and improvement of relevant seaweed species through omics.

Despite recent growth and investment in the U.S. aquaculture industry, several challenges still exist. Primarily, securing permits for coastal domestication is a significant bottleneck for those wishing to enter the industry (Kim et al., 2019). Offshore farming is considered a more suitable route for aquaculture as it presents fewer permitting conflicts (Buck, Krause, & Rosenthal, 2004; Cicin-Sain et al., 2001; Kim et al., 2019; Tiller, Gentry, & Richards, 2013). However, offshore farms are recognized as more stressful environments for macroalga due to stronger currents, limited nutrients, and cooler temperatures (Charrier, Wichard, & Reddy, 2018; Roleda & Hurd, 2019; Solan & Whiteley, 2016).

**FIGURE 1:**
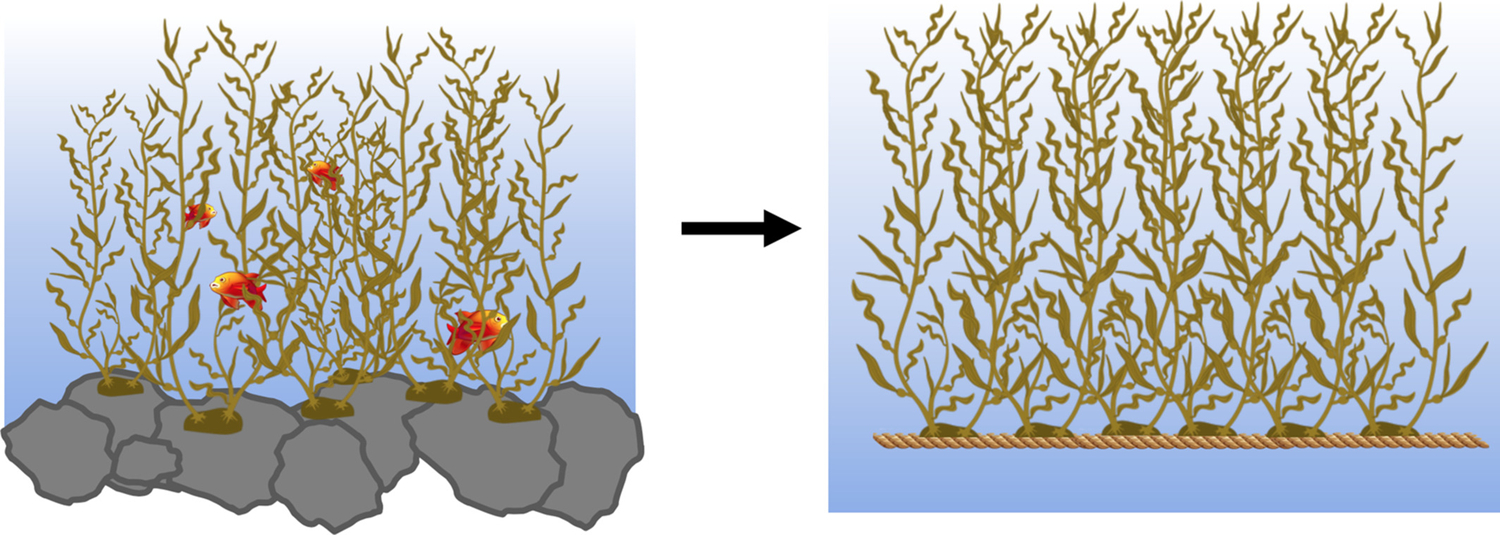
Domestication of marine macroalgae species. Many wild marine macroalgae populations settle on rocky substrates, providing important habitats for hundreds of marine species. There is much variation in rate of growth and chemical composition among wild macroalgae populations. Similar to agricultural crops, this natural phenotypic variation is reduced in domesticated macroalgae cultivars as the phenotype is optimized

Breeding programs can and have been developed to improve the productivity of seaweeds in certain environments or tailor crops to enhance specific characteristics of interest (Figure 1) (Hwang, Yotsukura, Pang, Su, & Shan, 2019; Walker, 2018; Zhang et al., 2016, 2018). Species of seaweeds targeted for this type of work include, but are not limited to, *Saccharina*, *Laminaria, Porphyra*, *Pyropia*, and *Macrocystis* (Kim et al., 2019). For example, in *Saccharina japonica*, QTL mapping has been used to identify QTLs responsible for higher yield by increasing blade size (Wang et al., 2018), and in *Saccharina latissima*, QTLs for stipe length were similarly identified (Mao et al., 2020). Macroalgal targeted breeding programs can be enhanced by more extensive incorporation of recent advances in multi-omics technology. This review makes the case for the inclusion of metabolomic and metagenomic data and analyses in continued efforts to domesticate and improve marine macroalgae for aquaculture.

## 2 | APPLICATION OF METABOLOMICS TO MACROALGA DOMESTICATION

Implicitly or explicitly, the metabolome—the suite of biological molecules present in a cell, tissue or organism—plays a large role in the domestication and improvement of crops (Burgess, Rankin, & Weidt, 2014; Dixon et al., 2006). Selecting for certain desired phenotypes is a necessary step in domestication to increase yield and cultivate optimal morphological characteristics. Investigating the genetics of cultivated crops has allowed the field of agriculture to create and employ improved crop varieties that have a higher yield (Murray et al., 2008; Wang et al., 2018) and better nutrient profiles or flavor (Gao et al., 2014), and to determine alleles involved in heterosis, allowing for the creation of crossing schemes in which offsprings perform far superior with respect to their parents (Huang et al., 2015). When combined with genomics and transcriptomics, metabolomics has further benefited agricultural plant domestication and improvement research in many areas including pesticide resistance (Aliferis & Chrysayi-Tokousbalides, 2011); microbial associations (Han & Micallef, 2016); crop flavor and nutrition profiles (Fernie & Schauer, 2009; Hall, Brouwer, & Fitzgerald, 2008); stress and heat tolerance (Che-Othman, Jacoby, Millar, & Taylor, 2020; Sung et al., 2015); and growth and yield (Obata et al., 2015).

### 2.1 | A need to adapt: global warming and resilient organisms

The projected global demand for food, livestock feeds, and bio-energy by 2050 will force the increased farming of low-carbon and carbon-sequestering marine resources, such as kelp and shellfish—especially in the face of large-scale environmental changes in climate and land use (Froehlich, Runge, Gentry, Gaines, & Halpern, 2018; Kim et al., 2019; Waite et al., 2014). The application of the suite of modern omics techniques, specifically genomics, transcriptomics, and metabolomics, to study and improve macroalgae is not only relevant to aquaculture seaweed production for human use; however, native macroalgae species are often habitat-forming and are integral species in coastal ecosystems (Teagle, Hawkins, Moore, & Smale, 2017). Wild temperate kelps have been increasingly threatened by the effects of global warming. Although globally marine macroalgae populations have been steadier than expected, when essential, habitat-forming macroalgae are displaced or lost in an environment, it can be extremely detrimental to the marine ecosystem (Krumhansl et al., 2016); the loss of *Macrocystis pyrifera* beds on Australian coastlines is one example (Mabin, Johnson, & Wright, 2019).

**FIGURE 2:**
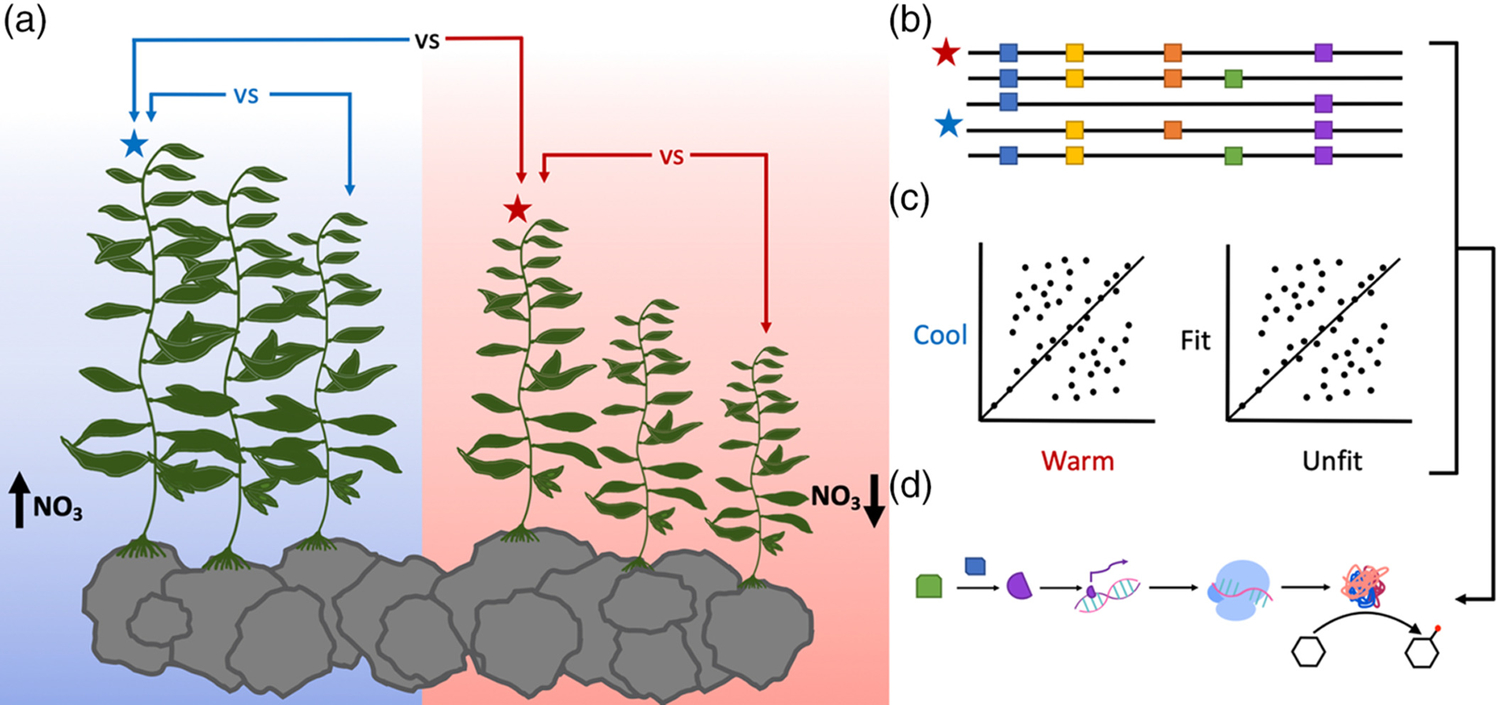
Use of omics techniques to investigate temperate kelp adaptation to climate change. (a) Kelps grown in cool water (blue, high [nitrate]) are expected to perform better overall than kelps grown in warmer water (red, low [nitrate]). The blue star denotes the hypothetical fittest cool-water individuals, while the red star is fittest in warm water. (b)–(d) Analyses of individual kelps in setup of (a). (b) Population genetics analysis of SNPs segregating between fittest kelps in cool versus warm water. (c) Differential expression analysis between fittest individuals in cool versus warm water. (d). Identification of pathways involved in kelp adaptation to warming oceans with genetic, transcriptomic, and metabolomic data

Several metabolomic studies to identify climate change-related responses have been conducted in plant species (Ahuja, de Vos, Bones, & Hall, 2010; Arbona, Manzi, de Ollas, & Gómez-Cadenas, 2013; Park, Seo, & Hegeman, 2014; Peñuelas et al., 2013). Recently, the use of metabolomics alongside other functional genomics techniques to identify metabolic responses in climate change-tolerant and construct conservation plans has been frequently suggested (De Ollas et al., 2019; Ouborg, Pertoldi, Loeschcke, Bijlsma, & Hedrick, 2010; Rilov et al., 2019). Climate change-resilient marine macroalgae (e.g., heat-, acidification-, low nutrient-tolerant) can be identified with analogous metabolomic studies to those proposed for terrestrial plants (Figure 2). The future of wild marine macroalgae populations as well as domesticated macroalgae cultivars will depend on biologically informed conservation and improvement efforts, which can be made more effective with metabolomic analyses.

### 2.2 | The metabolome of marine macroalgae

Metabolite profiling and analysis has a long history in marine macroalgae (Gupta, Thakur, Baghel, Reddy, & Jha, 2014), but has focused on metabolites that are bioactive, pharmaceutically relevant compounds (Davis & Vasanthi, 2011; Greff, Zubia, Payri, Thomas, & Perez, 2017); seasonal variation (Surget et al., 2017); delineating biochemical differences of green, red, and brown algae (Belghit et al., 2017); profiling during reproductive fragmentation (He et al., 2019); and stress, defense, and environmental responses (Gaubert, Payri, Vieira, Solanki, & Thomas, 2019; Gaubert, Rodolfo-Metalpa, Greff, Thomas, & Payri, 2020; La Barre, Weinberger, Kervarec, & Potin, 2004; Ritter et al., 2014). Domestication- and improvement-based metabolomic studies in macroalgae are sparse, even for model organisms.

A promising step forward in metabolomics of marine macroalgae was the development of a metabolomic “atlas” for brown macroalgae, called EctoGEM, generated through a genome-scale analysis of the metabolome of the model organism *Ectocarpus siliculosus* (Prigent et al., 2014). The state of omics research in marine macroalgae is advancing rapidly (Cock et al., 2010; Collén et al., 2013; Liu et al., 2019; Michel, Tonon, Scornet, Cock, & Kloareg, 2010; Ritter et al., 2014), and it is rising to meet more advanced functional genomics techniques to improve and optimize macroalgal species for aquaculture applications.

### 2.3 | Metabolomic analysis through a structural equation modeling framework

Metabolomic data sets are constructed using mass spectrometry (MS) and nuclear magnetic resonance (NMR) on tissues of interest (Emwas et al., 2019; Zacharias, Altenbuchinger, & Gronwald, 2018). The specific classes of primary and secondary metabolites captured by varying MS and NMR analyses have been reviewed extensively elsewhere (Bingol, 2018; Emwas, 2015; Emwas et al., 2019; Gupta et al., 2014; Kumar, Kuzhiumparambil, Pernice, Jiang, & Ralph, 2016; Nemkov, Reisz, Gehrke, Hansen, & D’Alessandro, 2019). Metabolites are the intermediate and final molecules modified and consumed by protein activity. The type and abundance of metabolites present are intimately tied to gene expression (Burgess et al., 2014; Nelson & Cox, 2017).

In an effort to investigate this relationship between metabolic flow and gene expression, metabolomic data sets are often complemented with RNA sequencing and phenotypic data (Fernie & Schauer, 2009). To model metabolic pathways with metabolomic and gene expression data, structural equation modeling (SEM), also known as confirmatory factor analysis, is a powerful tool. Modeling the metabolism provides a deeper understanding of the underlying pathways determining phenotypes of interest and how specific genetic variants perturb these metabolic pathways (Karns et al., 2013) SEM is a supervised approach based on geneticist Sewell Wright’s path analysis, where the order and direction of the relationships between genes and metabolites are intrinsic parts of the structural model (Igolkina & Samsonova, 2018; Wright, 1918, 1921). Previously, SEM has been used to model gene regulatory networks (GRNs) in both animals (Fear et al., 2015) and plants (Igolkina, Bazykin, Chizhevskaya, Provorov, & Andronov, 2019). Recent applications of SEM include annotations of underlying pathways for biomass development in rice (Momen, Campbell, Walia, & Morota, 2019), grain yield in wheat (Vargas, Crossa, Reynolds, Dhungana, & Eskridge, 2007), and body mass index in humans (Kaakinen et al., 2010). They have also been used to construct local GRNs based on patterns of differential gene expression (Aburatani, 2012; Tarone, McIntyre, Harshman, & Nuzhdin, 2012). The SEM framework can validate results using a variance × covariance structure of metabolites and transcripts across genotypes. This allows for the expansion of metabolic and transcriptomic networks. Using existing knowledge about a given GRN as a baseline model, geneticists can systematically scan macroalgae genomes for additional components and improve their understanding of existing networks. To validate constructed metabolic networks, flux balance analysis, a network reconstruction-based approach, is often used to model the flow of small molecules through known reactions (Orth, Thiele, & Palsson, 2010).

Many macroalgal systems, such as the model brown macroalga *E. siliculosus*, model red alga *Chondrus crispus*, or the commercial kelp *S. japonica*, have been characterized with genomic and transcriptomic methods (Cock et al., 2010; Collén et al., 2013; Liu et al., 2019). Expanding upon these data to develop full metabolomic models for organisms can reveal which genetic variants affect phenotype, which can be probed to maximize beneficial (e.g., growth and yield) phenotypes and would particularly benefit macroalgae crops in aquaculture.

As more studies harnessing modern omics methods are conducted in macroalgae, domestication and improvement of macroalgae species will become more efficient and be able to accomplish phenotypic changes with informed crossing schemes and genetic manipulation, which have taken thousands of years for traditional crop domestication in agriculture (Zsögön et al., 2018).

### 2.4 | The macroalgae holobiont: combining metabolomics and metagenomics to probe host–symbiont interactions

The term “holobiont” emerged in the context of holistic biology as early as the 1940s with Dr. Adolf Meyer-Abich (Baedke, Fábregas-Tejeda, & Nieves Delgado, 2020). Modern usage of the term to describe the relationship between organisms and their closely associated microbiota has been traced back to work by Dr. Lynn Margulis in 1991. Initially described as the relationship between a host and a single symbiont, the term has since evolved to describe a host organism and its associated biome, specifically including symbionts without which the holobiont would be able to perform its functions (Margulis, 1991). To learn more about the evolution of this term, we refer the reader to a recent review by Baedke et al. (2020). The holobiont concept has transformed the way scientists think about host organisms and their associated microbiota (collectively termed the microbiome). Specifically, it promotes the symbiotic relationship between host and microbiome by considering the microbiome and extended phenotype of the host and recognizing how both entities are influenced and shaped by the other. This frame of thought has been applied to microbial studies of many eukaryotes, including plants and mammals.

Applying this concept to seaweeds is a particularly strong example of holobionts in action, as microorganisms (specifically bacteria) are recognized to play an essential role in the health and fitness of aquatic plants and algae. As previously reviewed in Egan et al., 2013, many species of macroalgae rely on their associated microbes to provide essential nutrients (such as CO_2_ and fixed nitrogen) required for proper growth and development (Egan et al., 2013). To that end, several models have been proposed on how macroalgae may go so far as to “recruit” beneficial microbes by creating a desirable habitat by, for example, metabolite or chemical mediation (Saha & Weinberger, 2019). Given these tight associations, it is reasonable that macroalgal species and their microbial communities may have coevolved to rely on each other’s biological mechanisms and lose redundant gene pathways. Indeed, there exists an exciting opportunity to leverage existing knowledge of the macroalgal holobiont and apply tools from metabolomics, metagenomics, and transcriptomics to understand what genes are present and being expressed in the host and microbial metagenome (Egan, Thomas, & Kjelleberg, 2008). Exploiting the fruits of these discoveries—which may include identifying microbe species that serve essential functions for the macroalgae holobiont, determining growth-promoting nutrients at specific macroalgae life stages, and creating beneficial or protective microbial inoculants—will be essential for future genomic strategies for domestication and improvement of economically relevant macroalgae species.

## 3 | APPLICATION OF METAGENOMICS TO MACROALGA DOMESTICATION

High-throughput sequencing, such as shotgun metagenomics, is a powerful resource for understanding microbial communities (Olson et al., 2019; Sieber et al., 2018; Wilkins, Ettinger, Jospin, & Eisen, 2019). Advances in this field have enabled the discovery of novel genes and pathways that contribute to overall microbiome function. By combining metagenomics data from the native microbiome with physiological and genomic data from the host, we can investigate how the microbiome can be utilized to optimize host growth. There are several review papers on the use of metagenomic data to understand taxonomic composition and functional profile of the microbiome (Alves et al., 2018; Chiu & Miller, 2019; Gilbert & Dupont, 2011; Quince, Walker, Simpson, Loman, & Segata, 2017), and how these methods can benefit aquaculture (Martínez-Córdova, Emerenciano, Miranda-Baeza, & Martínez-Porchas, 2015; Tello et al., 2019). Specifically, functions of the microbiome can be exploited to improve crop productivity by providing essential nutrients or increasing disease resistance (Caruso, 2013; Ezemonye, Ogeleka, & Okieimen, 2009; Martínez-Córdova et al., 2015).

Application of these methods to macroalgae domestication is a unique opportunity and a powerful tool for research in agriculture and microbiomes because macroalgae is a fast-growing organism and its overall fitness is tightly intertwined with the native microbiome (Busetti, Maggs, & Gilmore, 2017; Egan et al., 2013; Florez, Camus, Hengst, & Buschmann, 2017; Laycock, 1974; Tello et al., 2019).

### 3.1 | Understanding the microbiome

One of the challenges around developing macroalgae as a commercial crop is that they are highly adapted to their local environment and do not demonstrate resilience or consistent physiological traits when farmed in new locations (Grebe, Byron, Gelais, Kotowicz, & Olson, 2019; Minich et al., 2018; Qiu et al., 2019; Zacher, Bernard, Daniel Moreno, & Bartsch, 2019). An example of this is giant kelp (*M. pyrifera*), a brown macroalgae that thrives in coastal environments and is a promising resource for future domestic biofuel production. Although giant kelp is one of the fastest growing organisms, farms along the US coast would not produce enough harvest to support giant kelp as a viable biofuel feedstock (Kim et al., 2019). Domestication of giant kelp in offshore farms would provide ample space for necessary production rates, but current practices of farming still remain uncompetitive to wild harvested macroalga. Similar challenges arise with the domestication of other species of macroalgae that are native to coastal environments. Offshore farms present a significant challenge in that they experience lower nutrient concentrations and are a more stressful environment for macroalgae compared to coastlines. Engineering solutions to overcome issues of containment, nutrient availability, and protection will be critical for offshore farming (Duarte et al., 2017; Roesijadi et al., 2008). Some individuals may be successful in this environment by chance, yet the unpredictable growth of seaweeds in offshore farms is a critical barrier to large-scale cultivation and commercial applications. Although breeding programs can be developed to optimize the gene content of macroalgae and promote genotypes that are resilient in low nutrient conditions, there remain significant challenges. However, limitations of breeding, such as trade-offs in yield and stress resistance, can be overcome by microbial treatments that improve crop fitness and predictable growth (Tack, Barkley, Rife, Poland, & Nalley, 2016). Development of these treatments requires better understanding of seaweed–bacterial associations, which are necessary for proper growth, recruitment, and development of seaweeds (Fitzpatrick et al., 2018; Morris et al., 2016).

The surface microbiome of seaweeds is composed of a diverse community of microorganisms that contribute to host health and form a biofilm across kelp blades. Macroalgal hosts rely on resident surface-associated microbes for proper growth and development. In addition to providing growth-benefitting compounds, epiphytic microbes provide a number of additional, beneficial services such as nutrient acquisition and protection from pathogens (Crawford & Clardy, 2011; Dubilier, Bergin, & Lott, 2008; Egan et al., 2008; Egan et al., 2013; Naragund et al., 2020; Wahl, Goecke, Labes, Dobretsov, & Weinberger, 2012). For example, certain seaweed-associated bacterial isolates are capable of fixing atmospheric nitrogen, which is often a limiting nutrient for kelp growth and development (Singh & Reddy, 2014).

Given that many species of macroalgae are not natively found offshore, this provides a unique challenge for open-ocean farming. The open-ocean environment constitutes a low nutrient and stressful environment for macroalgae (Hawkes & Connor, 2017). For field applications, microbial inoculations face an additional challenge as native members of the kelp microbiome may outcompete introduced microbes or their interactions may prove to be detrimental to kelp growth (Gause, 1934; Hutchinson, 1957; van Veen, van Overbeek, & van Elsas, 1997). By investigating community assembly patterns, we can navigate ecological niches to reduce competition and build a predictive understanding of how microbial treatments will impact the microbial community and overall growth of seaweed in aquaculture. Understanding functional traits across the community is also vital to developing successful microbial treatments for aquaculture. Although it remains a challenge to characterize function across the community, metagenomic sequencing summed across taxa provides sufficient representation of overall function compared to species-specific characteristics (Fierer, Barberán, & Laughlin, 2014; Jiang, Xiong, Danska, & Parkinson, 2016). Furthermore, host genotype also plays a key role in recruiting and sustaining a beneficial microbial community (Horton et al., 2014). By treating the microbiome as an extended phenotype of the host, one can perform a GWAS to identify genotypes or specific genes that are better suited to supporting a beneficial microbial community, which can guide future breeding design to compound the growth benefit of fast-growing genotypes and beneficial microbes (Awany et al., 2019).

Few studies have attempted to rigorously interrogate macroalgal host–microbe associations, assess the impact of host genotype on microbial community recruitment, or utilize these interactions to support macroalgae as a commercial crop. Amplicon sequencing, such as that of 16S rRNA, is a great resource for understanding species diversity in microbial communities. However, the added benefit of using metagenomic shotgun sequencing for studying the microbiome is that the greater genomic coverage and data output give insight into overall functional diversity, in addition to identifying unique and novel members of the community. Utilizing these types of data would enable us to characterize not only community assembly patterns and identify beneficial species of the microbiome, but also the impact of genetic diversity on seaweed-associated microbial communities to find host genes promoting or restricting recruitment of different symbionts. Although several studies have investigated the microbial community of several macroalgal species (Hawkes & Connor, 2017; Minich et al., 2018; Morris et al., 2016; Smith, Brzezinski, Melack, Miller, & Reed, 2018), this field would benefit from a large-scale study in a single well-controlled farm environment to understand microbiome structure and functional diversity in the context of a wide range of macroalgae genotypes. Understanding the mechanisms and functions of the microbial community, and the interaction between macroalgae and bacteria in offshore farms, is essential for ensuring success of the rising macroalgae aquaculture industry.

### 3.2 | Using metagenomic data to understand community assembly patterns of the microbiome

It is well recognized that targeted metagenomic sequencing, such as 16S rRNA sequencing, is frequently insufficient to characterize taxonomic and functional variation in microbial communities. Shotgun sequence metagenomic data can be used to fully annotate the species and functional diversity of microbial communities across a wide range of macroalgal species. Taxonomic identification and analysis of the microbial community with shotgun data requires the construction of Metagenomic Assembled Genomes. An example of a program to assist with this step is Anvi’o, an advanced analysis and visualization platform for omics data (Eren et al., 2015). To create consensus taxonomy for each unique genome, Anvi’o uses both single-copy core genes (SCGs) and the taxonomy determined by The Genome Taxonomy Database (Parks et al., 2018; Parks et al., 2020). Established species abundance tables then enable one to investigate community assembly patterns and develop a predictive understanding of how introduction of novel species will interact with the native community. A challenge often associated with analysis of metagenomic data is low coverage organisms and closely related taxa. To correct for this, there exist programs such as BinSanity, which uses an algorithm that clusters assemblies using coverage with compositional-based refinement to optimize bins containing multiple source organisms (Graham, Heidelberg, & Tully, 2017).

Previous studies have analyzed microbial and benthic macroinvertebrates using community ecology tools such as beta (*β*) and zeta (*ζ*) diversity (Doane, Haggerty, Kacev, Papudeshi, & Dinsdale, 2017; Simons, Mazor, Stein, & Nuzhdin, 2019). These tools, which are established methods for measuring compositional change across ecological communities, can be used to analyze community assembly patterns in the context of macroalgal surface microbiomes (Hui & McGeoch, 2014; McGeoch et al., 2019). These tools traditionally require that microbiome samples be collected from separate sites and use distance between sites to measure assembly patterns across a larger area. However, in macroalgal farm settings, by treating each individual as an independent community, and having information on the genome of the host, genetic distance between individuals can be used to establish the foundation for community assembly patterns. Moving from individual phenotypes (microbial loads) to functional groups, exploratory and confirmatory factor analyses, such as structural equation models described earlier, combined with QTL mapping can be used to determine how kelp genes affect community structures. These computational tools are innovative and have recently been applied to analyze the effects of human genetic variation on the metabolome (Igolkina & Samsonova, 2018), but have not yet been considered for microbial communities.

### 3.3 | Identifying beneficial bacteria using metagenomics and physiological crop traits

Metagenomic analysis in macroalgal farm settings provides a powerful tool when combined with physiological trait data of crops. By focusing on desired physiological traits, such as blade weight or nutrient composition, one can establish a metric to assess “successful” growth and consequently identify beneficial microbial species or groups of species. Co-occurrence networks can be used to determine microbial species most often associated with successful growth. These species can be considered “beneficial” and used to tailor future aspects of the analysis pipeline, such as analysis of functional traits. Understanding functional traits across the community is vital to developing successful microbial treatments for aquaculture. One trait of particular interest is nitrogen fixation. Nitrogen is an important resource for macroalgae growth (Hanisak, 1983; Harrison & Hurd, 2001; Zehr, Braun, Chen, & Mellon, 1996). Metagenomic sequence data would enable one to characterize symbiotic relationships between seaweed and nitrogen-fixing bacteria that have not yet been described. Preliminary studies have demonstrated a pattern of strong, and potentially vertical, co-transmission of *Mesorhizobium* spp. and *Sinorhizobium* spp. with giant kelp (Minich et al., 2018). This points to an opportunity to enhance nitrogen fixation in brown macroalgae and optimize growth since more than half of nitrogen fixation in *Sargassum* is thought to be derived from associated microbes (Phlips & Zeman, 1990; Raut, Morando, & Capone, 2018) and is known to be a limiting factor in *M. pyrifera* (Minich et al., 2018; Raut et al., 2018).

For this type of analysis, one can consider each genotype as an independent experimental observation with a unique effect on its associated microbiome. Specifically, metagenomic shotgun and 16S rRNA sequencing of the microbiome can be used in a quantitative model to infer kelp genes affecting recruitment and diversity of the native microbial communities. Community profiling, including species abundance and functional niches, can be assessed using established methods for taxonomy assignment and gene annotation (Eren et al., 2015; Ortiz-Estrada, Gollas-Galván, Martínez-Córdova, & Martínez-Porchas, 2019; Sieber et al., 2018; Vollmers, Wiegand, & Kaster, 2017; Woloszynek et al., 2019).

### 3.4 | Characterizing impact of host genotype on microbial community

Similar to work done in other organisms, structural equation models and QTL mapping can be used to determine how kelp genes affect overall community structure of the native microbiome (Chen et al., 2018; Horton et al., 2014; Jones, Garcia, Furches, Tuskan, & Jacobson, 2019). There have been a number of studies investigating the microbiome of several species of seaweeds such as *Porphyra* and *Pyropia* (Aydlett, 2019; Miranda, Hutchison, Grossman, & Brawley, 2013; Quigley, Morrison, Mendonça, & Brawley, 2018; Yan et al., 2019). The value of these studies, and overall impact on the macroalgae aquaculture industry, can be enhanced by further considering the impact of the most (macroalga) genotype on the recruitment of the native microbiome community structure.

For every group of kelp genotype with significant microbiome difference, one can generate co-occurrence networks to illustrate the likeliest associations between kelp genes of interest and particular members of their microbiome. In farm settings, unique kelp genotypes can be considered as independent experimental observations, each with a unique effect on its associated microbes. Similar work has been applied in humans (Igolkina et al., 2018), agriculture (Shin et al., 2019), and other aquaculture crops (Simons, Churches, & Nuzhdin, 2018). Metagenomic work for large numbers of samples can be cost prohibitive. Although a restricted number of metagenomic samples can be limiting for GWAS, this can be overcome by identifying strongly diverged populations of macroalgae and focusing on genotypes collected by hybrid zones. By having a sufficient number of samples come from hybrid zone lineages and focusing on QTL mapping, it becomes more feasible to have sufficient statistical power and replication to determine correlations between kelp genotypes and their microbiomes. To further focus this type of analysis, genotypes represented in this work, and microbiome samples to be analyzed, can be chosen based on whole genome sequencing (WGS) data from macroalgal individuals.

### 3.5 | Technical challenges of analyzing host genome impact on microbiome

To accomplish GWAS and QTL analysis, the microbial community should be treated as a multi-trait extended phenotype, with groups of community members or functions considered as different traits. For sparse data sets, pairwise distance matrices using beta-diversity can be used as the quantified host phenotype. Several approaches for multi-trait models have been proposed, but analysis can be challenging with correlated traits such as species abundance (Hackinger & Zeggini, 2017; Yang & Wang, 2012). One way to cope with correlated traits is to model the inter-trait covariance with random effect in linear mixed-effects models (Laird & Ware, 1982). Until recently, this model could use only a pair of correlated traits at a time due to the computational intensity (Korte et al., 2012). To reduce this load, variable reduction techniques have been suggested to replace several phenotypic traits with new independent constructs. These constructs play the role of new traits and can be obtained with a standard principal component analysis of traits, various principal components of heritability (Lange et al., 2004; Baltimore, 1986; Wang, Fang, & Jin, 2007) or pseudo-principal components (Gao et al., 2014). Another challenge in association studies is to develop a powerful multi-locus model. Testing SNP by SNP, single-locus models require correction for multiple testing afterward, which can eliminate important quantitative trait variants. To avoid this problem, multi-locus models, that consider all markers simultaneously, can be applied. Due to the “large *p* (number of SNPs), small *n* (sample size)” problem, many multi-locus models are based on regularization/penalized techniques including LASSO (Wu, Chen, Hastie, Sobel, & Lange, 2009), elastic net (Cho, Kim, Oh, Kim, & Park, 2009), Bayesian LASSO (Yi & Xu, 2008), and adaptive mixed LASSO (D. Wang, Eskridge, & Crossa, 2011). Other multi-locus methods (incorporated in the mrMLM package) involve two-step algorithms, which first selects candidate variants in single-locus design and then examines them together in a multi-locus manner (Wen et al., 2017).

Despite the broad spectrum of multi-trait and multi-locus models in GWAS and trait prediction studies, only a few of them simultaneously incorporate correlated traits and several associated variants (Dutta et al., 2018; Lippert, Casale, Rakitsch, & Stegle, 2014; Liu et al., 2016; Weighill et al., 2019; Zhan et al., 2017). In principle, multi-trait and multi-locus models have a potential to reveal complex and important types of associations, for instance, a single variant might have a direct effect on one trait, and an indirect impact on the other trait; an SNP may act on a single trait or its effect might be pleiotropic affecting several traits. However, none of these trait–variant associations are explicitly embedded into known models, but they can be directly accounted for with the previously described method SEM, a multivariate statistical analysis technique first introduced for path analysis by geneticist Sewell Wright (Wright, 1918, 1921). SEM has been widely used in the fields of genetics, econometrics, and sociology, and current SEM applications are gradually shifting to molecular biology (Igolkina et al., 2018). SEM models have also been applied in the association studies in both multi-trait and multi-locus designs. For example, the GW-SEM method was developed to test the association of a SNP with multiple phenotypes through a latent construct (Verhulst, Maes, & Neale, 2017). It was demonstrated that in comparison with the existing multi-trait single-locus GWAS software package GEMMA (Zhou & Stephens, 2012), GW-SEM provides for more accurate estimates of associations; however, GEMMA was almost three times faster than GW-SEM. Another SEM-based model that can be used in association studies was proposed for multi-trait QTL mapping (Mi et al., 2010). This method proposes that phenotypes are causally related, forming a core structure without latent constructs, and that QTLs play the roles of exogenous variables to the structure. This approach allows the model to decompose QTL effects into direct, indirect, and total effects.

Addressing these challenges is critical to understanding and properly analyzing the impact of the host genome on the recruitment of the native microbiome. Applications described here have not been applied to the seaweed microbiome and should be explored in more detail to have an impact on the aquaculture industry.

## 4 | CONCLUSIONS

Advances in modern omics technology, including in sequencing and metabolite profiling, have created powerful tools and analysis pipelines for understanding the metagenome and metabolome of living organisms. Established pipelines from functional genomics research in agriculture are beginning to be applied to seaweed systems but must be expanded to improve the development of seaweed aquaculture. Metabolomic analysis has been used to fine-tune domestication and crop improvement strategies in agriculture by revealing pathways involved in crop nutrition, flavor, and stress response. Applying metabolomics analyses—alongside other functional genomics analyses—more broadly in marine macroalgae toward improvement of cultivars for aquaculture will allow scientists to identify significant pathways for macroalgae domestication more efficiently.

The microbiome plays an important role in the overall health of several macroalgal species and it is imperative to further understand microbial communities in the context of host genotypes to ensure the success of the rising macroalgae industry in the United States. Development and understanding of host–microbe associations in aquaculture can lead to microbial inoculations to boost crop yields, and future breeding programs should focus on the compounded benefit of seaweed genotypes and optimized microbial communities. Further investigation of the microbiome and metabolome in seaweeds has the potential to greatly improve current methods of US macroalgae domestication.

By treating the metabolome and microbiome as extended phenotypes, pathways for domestication and optimized breeding go beyond the traditional desired physiological traits in crops, such as size and nutrient load. Specifically, functional traits of the microbiome, such as its ability to fix nitrogen or prevent disease, can be exploited to improve overall crop health and fitness. Application of these tools in macroalgae is a unique opportunity because it is a fast-growing organism that heavily relies on an optimal, environment-adapted metabolic profile, and beneficial microbial interactions.

**FIGURE 3:**
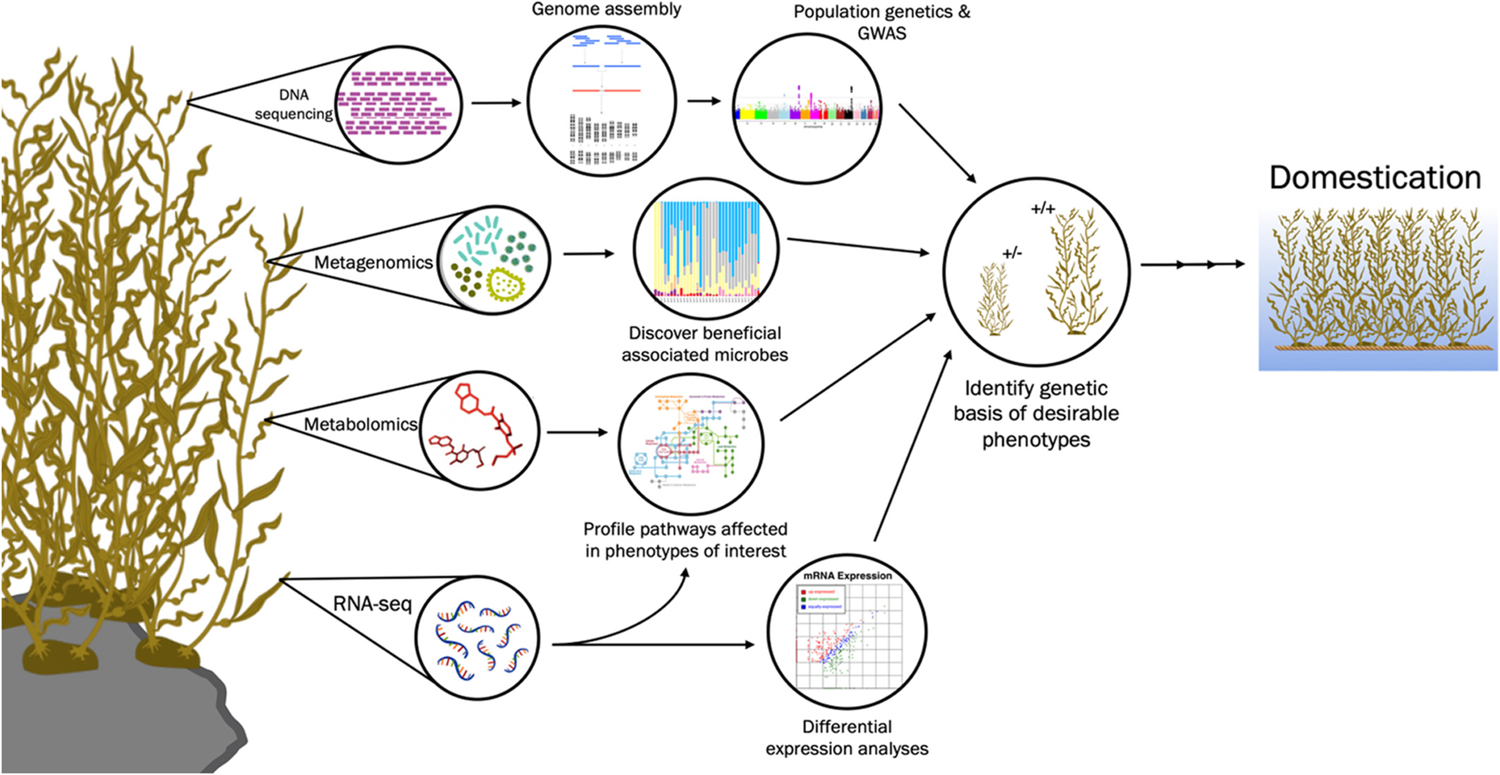
Overview of omics techniques currently used and proposed for use in macroalgae domestication and improvement. Proposed schema for the application of DNA and RNA sequencing, metagenomics to efficiently improve and domesticate macroalgae, emphasizing how each analysis can complement and build upon others. The information gained through performing metagenomic and metabolomic analyses on top of conventional sequencing analyses—including the identification of potential microbial inoculants for macroalgae cultivars, essential nutrients for macroalgae growth, and the mechanism by which genetic variants of macroalgae species produce desirable phenotypes—makes a case for the increased collection and utilization of these important data sets and analyses

With the rise of marine macroalgae in the aquaculture industry, there exists a unique opportunity to utilize modern developed omics tools to domesticate macroalgae species rapidly, including by optimizing their metabolomic and metagenomic profiles (Figure 3). As future breeding programs are developed to establish the United States as a world leader in aquaculture, we must incorporate the powerful tools and recent advances in modern omics techniques to guide our breeding protocol and turn to functional genomics to greatly improve upon traditional breeding programs. Metagenomics and metabolomics analyses provide important contributions that deserves future consideration as scientists continue to explore omics in aquaculture.
